# Supplementary material for: Complexome profiling of the Chlamydomonas psb28 mutant reveals TEF5 as an early PSII assembly factor
Source: Plant Cell. 2025 Mar 17;37(6):koaf055. doi: 10.1093/plcell/koaf055 (PMC12164590; doi:10.1093/plcell/koaf055)
Supplement: koaf055_Supplementary_Data [file koaf055_supplementary_data.zip › psb28_interactive.zip!/plots/intensity/qualityPlots/peptides/MME1_Cre06.g268750.t1.2.html]

### Cre06.g268750.t1.2

Trivial name: MME1  
SeqCoverage [median]: 26.40  
Mass: 69.88 kDa  
  
 

| Sequence | MissedCleavage | Mass [Da] | wtIntensity | muIntensity | Score |
| --- | --- | --- | --- | --- | --- |
| ADPTHEGEDLLAVVR | 0 | 1620.81 | 888.84 | 10093.46 | 76.59 |
| AIFASGSPQPPVTLASGR | 0 | 1754.93 | 2700.05 | 4100.61 | 151.78 |
| FMEDYYQPK | 0 | 1219.52 | 0.00 | 2804.93 | 114.28 |
| LFTEEVLAAMNK | 0 | 1364.70 | 0.00 | 557.95 | 99.65 |
| LGGVTSAMAR | 0 | 961.50 | 4006.30 | 7028.40 | 137.14 |
| LIPEEELER | 0 | 1126.59 | 1401.88 | 5258.06 | 92.44 |
| LLQELQDRNETLFYR | 1 | 1937.00 | 665.87 | 7227.33 | 88.97 |
| MIASGMQK | 0 | 864.42 | 747.46 | 1770.68 | 62.72 |
| MLMAAAEELPR | 0 | 1230.61 | 1959.52 | 5156.61 | 116.30 |
| NETLFYR | 0 | 941.46 | 0.00 | 446.96 | 70.91 |
| SLTMELQAAR | 0 | 1118.58 | 2200.93 | 8917.18 | 116.30 |
| TNIADFAKPFAR | 1 | 1349.71 | 1110.43 | 12986.17 | 81.86 |
| VKPTILLGLAGAGR | 1 | 1364.85 | 1329.01 | 26926.10 | 103.97 |
| VLPVVIDVGTNNEALR | 0 | 1707.95 | 2058.78 | 0.00 | 128.85 |
| YAQGDEALR | 0 | 1021.48 | 1790.03 | 3727.39 | 102.51 |
|  |  |  | 20859.1 | 97001.8 |  |
